# Impact of emerging virus pandemics on cause-specific maternal mortality time series: a population-based natural experiment using national vital statistics, Argentina 1980-2017

**DOI:** 10.1016/j.lana.2021.100116

**Published:** 2021-11-19

**Authors:** María Elena Critto, Yordanis Enriquez, Miguel Bravo, Lenin de Janon Quevedo, Ruth Weinberg, Adolfo Etchegaray, Elard S. Koch

**Affiliations:** a Division of Epidemiology, MELISA Institute, Concepción, Chile; b Programa de Doctorado en Sociología, Facultad de Ciencias Sociales, Pontificia Universidad Católica de Argentina, Ciudad de Buenos Aires, Argentina; c Facultad de Ciencias de la Salud, Universidad Católica Sedes Sapientiae, Lima, Perú; d School of Public Health, Faculty of Medicine, University of Chile, Santiago, Chile; e Facultad de Ciencias Médicas, Pontificia Universidad Católica de Argentina, Ciudad de Buenos Aires, Argentina; f Facultad de Medicina, Universidad de Buenos Aires, Buenos Aires, Argentina; g Hospital Universitario Austral, Facultad de Medicina, Universidad Austral, Buenos Aires, Argentina

**Keywords:** MMR, maternal mortality ratio, ITS, Interrupted Time Series, A[H1N1]pmd09, H1N1 pandemic A influenza virus 2009, WHO, World Health Organization, ICD, International Classification of Diseases, APC, annual percentage change, AAPC, average annual percentage change, Maternal Mortality, Public Health Surveillance, H1N1 Virus, Pandemics, Interrupted Time Series Analysis, Argentina

## Abstract

**Background:**

Emerging pandemic viruses may have multiple deleterious effects on maternal health. This study examines the effects of a pandemic influenza virus on cause-specific maternal mortality time series, using Argentinian vital statistics.

**Methods:**

We conducted a population-based natural experiment from national vital records of maternal deaths between 1980 and 2017. Joinpoint regression models were used to model time series of the maternal mortality ratio (MMR). The sensitivity of the registry to detect the effects of the pandemic H1N1 2009 influenza virus on cause-specific MMR was analysed using a panel of parallel interrupted time series (ITS).

**Findings:**

Over this 38-year study, the MMR decreased by 58·6% (69·5 to 28·8 deaths/100,000 live births), transitioning from direct obstetric causes (67·0 to 21·1/100,000 live births; 68·4% decrease) to indirect causes (2·6 to 7·7/100,000 live births; 196·2% increase). The regression analysis showed an average reduction of -2·2%/year (95% CI: -2·9 to -1·4) with 2 join points in the total trend (1998 and 2009). Parallel ITS analyses revealed the pandemic H1N1 virus had an increasing effect on mortality from the respiratory system- and sepsis-related complications (level change 4·7 and 1·6/100,000 live births respectively), reversing after the outbreak. No effect was found on MMR from hypertensive disorders, haemorrhage, abortive outcomes, other direct obstetric causes, and indirect non-respiratory comorbidities.

**Interpretation:**

The Argentinian maternal death registry appears sensitive to detect different effects of emerging infectious epidemics on maternal health. In a population-based natural experiment, pandemic H1N1 virus impacted maternal mortality almost exclusively from the respiratory system- and sepsis-related complications.

**Funding:**

Supported by FISAR www.fisarchile.org

**Research in context**

**Evidence before this study**

PubMed and Medline were searched using the terms "H1N1" and "interrupted time series" and "maternal mortality", in English and Spanish, for articles published up until June 8, 2021. In addition, because of the ongoing coronavirus pandemic, we include the terms “SARS-CoV-2” and “COVID-19” in our search. In the context of epidemiological surveillance of emerging pandemic pathogens such as the former H1N1 2009 influenza virus, or the current SARS-CoV-2 (viral agent for COVID-19), it is critically relevant to know the sensitivity of vital records of live births and maternal deaths to explore potential deleterious effects of a new pathogen on specific organs or systems, stages of pregnancy, comorbidities, and ultimately, on the offspring. Over the last two decades, the quality of national mortality statistics improved in most countries, showing an important advance in reducing maternal deaths globally. However, there is a lack of information about whether this improvement has translated into greater capacity or sensitivity of national registries to detect specific effects of exogenous factors such as global epidemics on maternal mortality trends. Although there are case-control, cross-sectional, and short in-hospital time series studies that describe an increase in maternal deaths during the H1N1 2009 pandemic for example, there is no available evidence of the use of population-based natural experiments to detect multiple effects triggered by this and other pandemic pathogens on specific causes of death on women during pregnancy, childbirth, and the puerperium.

**Added value of this study**

Before the current public health crisis triggered by SARS-CoV-2, the magnitude of the global pandemic caused by H1N1, offers the most recent epidemiological experience to explore the ability of vital records to detect specific effects of an emerging pathogen in large time series of maternal mortality. For the first time, we took advantage of the pandemic H1N1 2009 influenza virus to design a population-based natural experiment and assess multiple effects on 38-year time series of total and cause-specific maternal mortality from Argentinian national vital statistics of live births and deaths of women during pregnancy, childbirth, and the puerperium. A panel of parallel interrupted time series (ITS) was used to assess the sensitivity of vital records, revealing very specific changes in maternal mortality trends triggered by the pandemic influenza virus.

**Implications of all the available evidence**

Argentinian national vital records of live births and maternal deaths provide an excellent example for the progress in reducing maternal mortality in Latin America over the past 38 years, transitioning from direct obstetric causes to indirect comorbidities unrelated to pregnancy. This country also appears to represent an interesting example for the improvement in the sensitivity of the maternal death registry to illustrate how an emerging pandemic virus may impact maternal health. In fact, in a population-based natural experiment with virtually complete time series of vital statistics, an abrupt interruption in the downward trend of the total maternal mortality was corroborated in 2009. Parallel ITS analyses revealed that the harmful effect of the pandemic H1N1 2009 influenza virus on maternal health, was remarkably restricted to respiratory system- and sepsis-related complications. Maternal mortality from non-respiratory comorbidities unrelated to pregnancy along other multiple obstetric causes were virtually unaffected. This evidence is compelling and relevant to comparatively contrast specific effects of the ongoing SARS-CoV-2 pandemic or any other emerging infectious global outbreak on maternal health in the future. Finally, major efforts will be needed in early organised healthcare to prevent maternal deaths and monitor the impact of emerging pandemic viruses on women's health during pregnancy, childbirth, and the puerperium.

Alt-text: Unlabelled box

## Introduction

Mortality data on a global scale reflect important advances in the health and life expectancy of the population during the last 50 years. Nonetheless, premature deaths by avoidable causes, such as maternal deaths, remain issues of global public health concern.^1^^,^^2^ Currently, there is a consensus on the importance of national vital statistics and epidemiologic surveillance data useful for conducting population-based natural experiments that allow monitoring the impact of exogenous factors or interventions on health.^3^ A natural experiment is a research design that allows the evaluation of the exposure to an event of interest that has not been manipulated by the researcher, and it is often recommended for evaluating the impact of public policies on population-level results, social disparities, and the deleterious effects of natural disasters, including epidemics.^4^

Emerging infectious epidemics may have multiple deleterious effects on women's health during pregnancy, childbirth, and the puerperium. For the epidemiological surveillance of new pandemic pathogens such as the former H1N1 2009 influenza virus, or the current SARS-CoV-2 (viral agent for COVID-19), it is critically important to know the sensitivity of maternal death records for detecting potential harmful effects on specific organs or systems, stages of pregnancy, comorbidities, and ultimately, on the offspring. However, although maternal mortality records improved in most countries over the last decades^1^, it is not immediately clear whether this improvement translated into greater sensitivity to detect specific effects of pandemics triggered by new pathogens.

The natural experiment design may be a useful and relatively simple method to monitor the quality of national vital records regarding its sensitivity to detect changes in mortality before and after the occurrence of an emerging infectious outbreak. In 2009 for example, the H1N1 influenza virus (hereafter A[H1N1]pmd09)^5^ spread rapidly around the world, seriously affecting certain vulnerable groups. A higher risk of maternal death was reported in epidemiological surveillance records during the outbreak in several countries.^6^, ^7^, ^8^, ^9^, ^10^, ^11^ Before the current public health crisis triggered by SARS-CoV-2, the magnitude of the global pandemic caused by H1N1, offers the most recent experience to assess the ability of vital records to detect specific effects of an emerging pathogen in large time series of live births and maternal deaths.

In Argentina, although maternal mortality prevention is a priority in public health policies and research agendas,^12^ there is a lack of information about the performance of national official mortality records to identify the potentially deleterious effects of emerging infectious epidemics on women's health. In this study, the sensitivity of Argentinian vital records of maternal deaths and live births were evaluated through a natural experiment design to explore the specific effects of the pandemic H1N1 2009 influenza virus during pregnancy, childbirth, and the puerperium. The objective was to model trends in maternal mortality between 1980 and 2017 and determine whether the pandemic influenza virus impacted on specific causes of maternal death.

## Methods

### Design

From official Argentinian national vital statistics, a population-based natural experiment^4^ was designed using parallel interrupted time series (ITS). The ITS is useful to evaluate public health interventions and natural events by using pre-/post-comparisons of a group over multiple time points to control for underlying trends.^13^ A systematic search was conducted of National Board of Health Statistics of the Ministry of Health data to collect the official audited records of live births and deaths of women during pregnancy, childbirth, and the puerperium between 1980 and 2017.^12^ The 1980-2005 data was formally requested from National Board of Health Statistics of the Ministry of Health. Over the study period, the Argentinian civil registration of maternal mortality data met standard comparisons at an international level^12^ by using the WHO International Statistical Classification of Diseases and Related Health Problems (ICD) - version 10 from 1997 to 2017, and version 9 from 1980 to 1996.^12^

### Maternal deaths

Maternal mortality was defined according to the WHO as “maternal death during pregnancy and childbirth or within 42 days of termination of pregnancy.[12]” Maternal deaths are classified into two main groups: direct obstetric causes and indirect obstetric causes, the latter considering all comorbidities unrelated to pregnancy such as cardiovascular diseases, diabetes, cancer, renal failure, and other chronic conditions.^12^ This study used the structure of the basic list of ICD-categorised causes along with a translator provided by WHO to assign equivalent codes based on the ICD-9 and ICD-10 versions (*Supplementary Table S1*). Four sources were considered for the construction of the cause-specific maternal mortality groups (*Supplementary Table S2*). Additional details about the ICD homologation carried out for this study are provided in the supplementary methods.

### Study variables

The dependent variables correspond to the total and cause-specific maternal mortality ratios (MMRs), which are calculated as the ratio between the number of maternal deaths and live births at the national level multiplied by 100,000 per year.^12^ The total MMR considers all maternal death causes in the ICD-9 (codes 630-676) and ICD-10 (codes O00-O99), excluding late maternal mortality (codes O96-O97).^12^ In addition, the specific MMRs of pregnancy with abortive outcome, sepsis, haemorrhage, hypertension, other direct causes, and indirect causes or comorbidities unrelated to pregnancy were computed. Similarly, the group of deaths from indirect obstetric causes were stratified to identify a subgroup of maternal deaths related to the respiratory system (ICD-9 code 648.9 and ICD-10 code O99.5). This group was labelled respiratory causes. The other indirect causes (ICD-9 codes 647-648 and ICD-10 O98-O99 codes excluding codes ICD-9 648.9 and ICD-10 O99.5) constituted the subgroup of all non-respiratory comorbidities unrelated to pregnancy. Finally, the year in which A(H1N1)pmd09 occurred was considered as an independent variable.

### Statistical analysis

Parallel time series were constructed for all time-dependent variables recorded between 1980 and 2017. Kolmogorov-Smirnov test was used to test the normal distribution assumption. For the analysis of MMR trends, a linear logarithmic regression was conducted through Joinpoint Regression Program version 4.8.0.1 (Surveillance Research Program, National Cancer Institute) to identify the number and place of the Joinpoints that represent the moments of trend change; the estimated annual percentage change (APC) of the dependent variable for each time segment; and the average annual percentage change (AAPC) in the whole period studied. The models were as follows^14^:

First, log (*Ry*) = *b_0_* + *b_1_y*, where log (*Ry*) is the natural log of the rate in year *y.*

$$The\;APC\;from\;year\;y\;to\;year\;y\;+1=\left[\frac{R_{y+1}\;-\;R_{y}}{R_{y}}\right]x100$$

$$=\frac{\left\{e^{b_{0}+b_{1}\left(y+1\right)}-e^{b_{0}+b_{1}\left(y\right)}\right\}}{e^{b_{0}+b_{1}\left(y\right)}}x100$$

$$=\left(e^{b_{1}}-1\right)x100$$

Second, if the slope coefficients for each segment are denoted as *b_i_s* and *w_i_s* is the length of each segment, then we obtain

$APC_{i}=\left\{\exp(b_{i})-1\right\}x100$ then:

$$AAPC=\left\{\exp\left(\frac{\sum w_{i}b_{i}}{\sum w_{i}}\right)-1\right\}x100$$

The accuracy of the data registry to reflect changes regarding the event of interest was evaluated by using an interrupted time series (ITS) approach in parallel cause-specific mortality trends. The ITS regression model^15^ was specified according to the equation:

$$Y_{t}=\beta_{0}+\beta_{1}T+\beta_{2}X_{t}+\beta_{3}X_{t}T_{t}+\in_{t}$$

In this model, *Y_t_* is a continuous measured outcome (i.e., the MMR), at regular intervals at each time point (*t*); *β_0_* represents a constant or starting point for the outcome; *β_1_* is the change in the outcome associated with a time unit increase *T_t_* which is interpreted as the underlying pre-intervention trend; *β*_2_ is the level of immediate change upon intervention *X_t_* which is specified as a dummy variable (pre-intervention period = 0 and post-intervention = 1); and β*_3_* indicates the slope change following the intervention regarding the initial trend. The ITS model considers the emergence of a natural event such as an A(H1N1)pmd09 outbreak ^11^ and evaluates trends and significant immediate changes or effect sizes attributable to pandemic H1N1 2009 virus with the linear least squares method. Coefficients with Newey-West standard errors were estimated to evaluate self-correlation and possible heteroscedasticity. All measures were computed with 95% confidence intervals (95% CI).

### Ethics Approval

This study used aggregated public data anonymised in the source, and it was approved by an institutional review board from Pontificia Universidad Católica Argentina.

### Role of the funding source

The funder of the study had no role in the decision for publication, study design, data collection, data analysis, data interpretation, or writing of the report.

## Results

**Figure 1:**
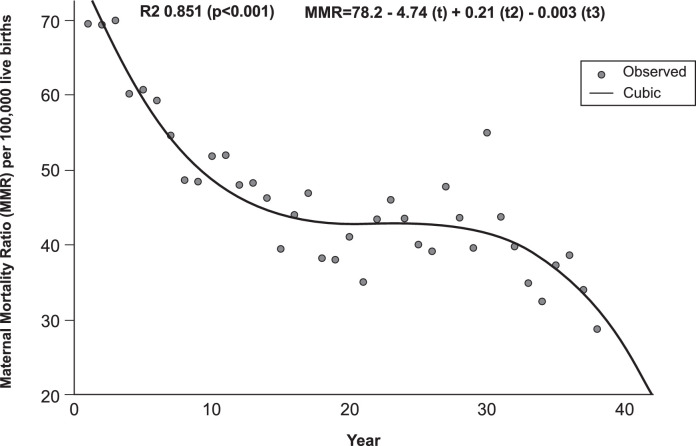
The best estimated curve of the total maternal mortality ratio (MMR) in Argentina for the period 1980-2017 through curvilinear regression analyses.

During the 38-year study period, 12,223 maternal deaths and 26,566,869 live births were officially recorded in Argentina, corresponding to an MMR of 46/100,000 live births. The total MMR decreased from 69·5 to 28·8 maternal deaths/100,000 live births (58·6% reduction). The best-estimated curve was cube-shaped with a goodness-of-fit of 85·1% (Figure 1).

The MMR related to abortive outcome had the largest decrease (24·5 to 4·3/100,000 live births, -82·6%), followed by a decrease in haemorrhage-related MMR (10·2 to 2·3/100,000 live births, -77·7%) (*Supplementary Table S3*). In contrast, the MMR attributable to indirect causes grouping all comorbidities unrelated to pregnancy increased from 2·6 to 7·7/100,000 live births (197%). The MMR specifically related to respiratory causes increased from fewer than 1/100,000 in 1990 to 2/100,000 live births in 2017. The year 2009 stands out because the highest MMR attributable to respiratory causes was found and was almost 10-fold the number of deaths in the previous year (12·7/100,000 live births vs 1·3/100,000 live births, respectively), contributing to 23% of the total maternal deaths (*Supplementary Figure S1*).

### Joinpoint regression models

**Figure 2:**
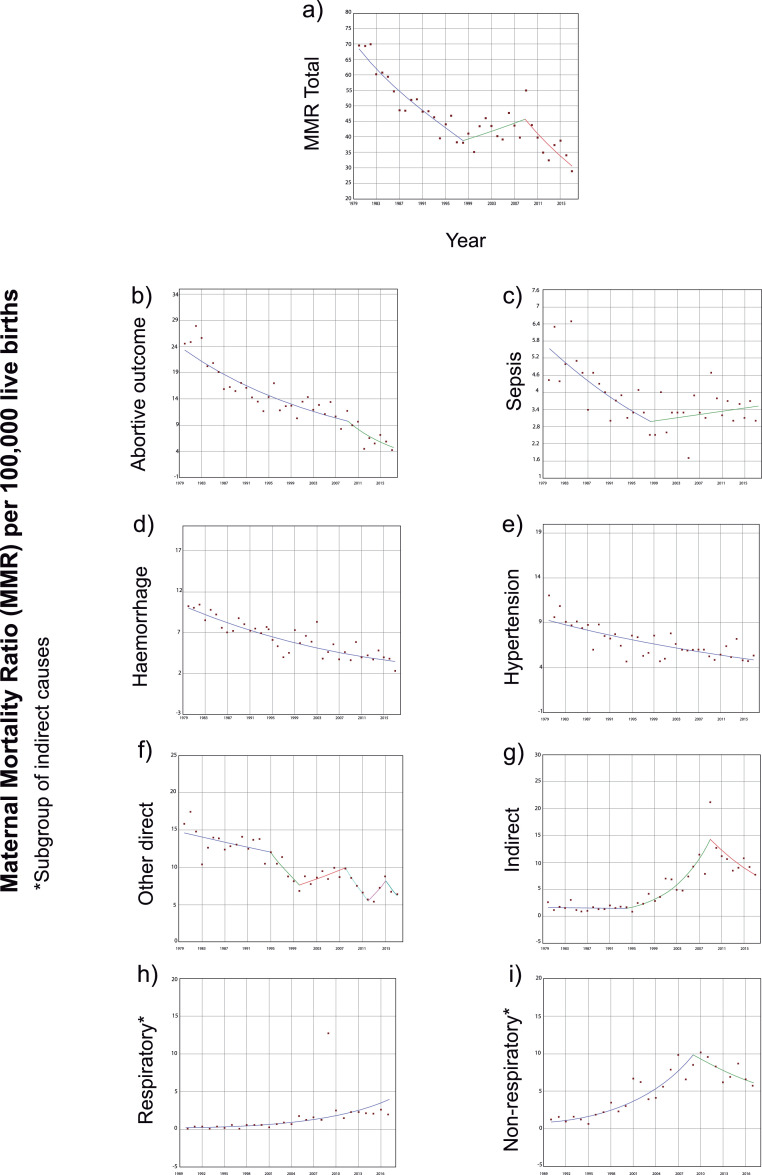
Linear logarithmic regression analysis (Joint point) for the identification of different segments of total maternal mortality ratio (a) and maternal mortality ratios due to abortive outcome, sepsis, haemorrhage, hypertension, other direct causes, and indirect causes (b to i), in Argentina, 1980-2017. Each linear segment is represented by a different colour.

The total and cause-specific MMR trends along with joinpoint regression results are detailed in Table 1, and segments are plotted in Figure 2. A join point in the curve was observed in 1998 and a second in the total MMR in 2009, limiting an intervening stagnant period. A downward trend was observed from 1980 to 1998, with an APC decrease of -3·11% (95% CI: -3·8 to -2·4) each year. From 2009, the downward trend re-accelerated to -4·86%/year (95% CI: -7·2 to -2·4) until 2017. The AAPC of the total study period was -2·2%/year (95% CI: -2·9 to -1·4).

**Table 1:** Linear logarithmic regression analyses to model trends and joinpoints for total MMR and cause specific MMRs due to abortive outcome, sepsis, haemorrhage, hypertension, other direct causes, and indirect causes, Argentina, 1980-2017

		**Years**	APC*	**p-value**	**95% CI**§	**Joinpoint**	**95% CI**
**MMR total**†							
	Trend 1	1980-1998	-3·11	<0·001	-3·8 - -2·4		
	Trend 2	1998-2009	1·49	0·106	-0·3-3·3	1998	1992-2002
	Trend 3	2009-2017	-4·86	<0·001	-7·2 - -2·4	2009	2005-2012
	AAPC⁎⁎	1980-2017	-2·2	<0·001	-2·9 - -1·4		
**MMR by abortive outcome**							
	Trend 1	1980-2009	-2·99	<0·001	-3·7 - -2·3	2009	2003-2012
	Trend 2	2009-2017	-8·58	0·001	-13·2- -3·7		
	AAPC	1980-2017	-4·2	<0·001	-5·4 - -3·1		
**MMR by sepsis**							
	Trend 1	1980-1998	-3·36	<0·001	-5·0 - -1·6	1998	1990-2006
	Trend 2	1998-2017	0·81	0·319	-0·8-2·5		
	AAPC	1980-2017	-1·2	0·035	-2·4 - -0·1		
**MMR by haemorrhage**							
	Trend 1	1980-2017	-2·82	<0·001	-3·4 - -2·3	··	··
	AAPC	1980-2017	-2·8	<0·001	-3·4 - -2·3		
**MMR by hypertension**							
	Trend 1	1980-2017	-1·72	<0·001	-2·2 - -1·2	··	··
	AAPC	1980-2017	-1·7	<0·001	-2·2 - -1·2		
**MMR by other direct causes**							
	Trend 1	1980-1995	-1·30	0·060	-2·6-0·1		
	Trend 2	1995-2000	-8·75	0·077	-17·6-1·1	1995	1982-2000
	Trend 3	2000-2008	3·34	0·130	-1·0-7·9	2000	1985-2006
	Trend 4	2008-2012	-13·90	0·068	-26·8-1·2	2008	1991-2009
	Trend 5	2012-2015	14·57	0·392	-17·1-58·4	2012	1999-2012
	Trend 6	2015-2017	-12·90	0·385	-37·0-20·4	2015	2002-2015
	AAPC	1980-2017	-2·3	0·230	-5·9-1·5		
**MMR by indirect causes**							
	Trend 1	1980-1994	-0·70	0·744	-4·9-3·7		
	Trend 2	1994-2009	16·44	<0·001	11·5-21·6	1994	1985-1997
	Trend 3	2009-2017	-7·31	0·135	-16·2-2·5	2009	2000-2013
	AAPC	1980-2017	4·4	0·007	1·2-7·7		
**MMR by respiratory indirect causes**‡							
	Trend 1	1990-2017	12·10	<0·001	8·6-15·7	··	··
	AAPC	1990-2017	12·1	<0·001	8·6-15·7		
**MMR by non-respiratory indirect causes**‡							
	Trend 1	1990-2009	13·19	<0·001	10-16·5	2009	2004-2013
	Trend 2	2009-2017	-5·68	0·268	-15·2-4·9	··	··
	AAPC	1990-2017	7·2	<0·001	3·5-11·1		

⁎ APC: Annual Percent Change;

§ CI: Confidence Interval;

† MMR: Maternal Mortality Ratio per 100,000 live births;

⁎⁎ AAPC: Average Annual Percent Change;

‡ Subgroup of indirect causes.

The MMR related to abortive outcomes showed a join point in 2009 (Figure 2b). In the first segment, between 1980 and 2009, the annual reduction was -3%/year. In the second segment, between 2009 and 2017, the reduction accelerated to -8·58%/year. The AAPC estimate was -4·2% (95% CI: -5·4 to -3·1) and almost doubled the observed reduction in total MMR.

The MMR attributable to complications by sepsis exhibited a downward trend of -3·36% (95% CI: -5·0 to -1·6) each year from 1980 to 1998, followed by a change in the slope, reflecting a stagnant period (Figure 2c). The AAPC indicated a total downward trend of -1·2% (95% CI: -2·4 to -0·1). The MMR attributed to haemorrhage, hypertension, and other direct causes (Figure 2d-f) showed downward trends but no changes in slopes or join points in 2009.

Two joinpoints were observed for indirect causes in the MMR corresponding to 1994 and 2009, limiting an intervening segment with an accelerated uptrend of 16·44%/year (95% CI: 11·5 to 21·6) (Figure 2g). The AAPC for the entire analysed period reflected an increase of 4·4% (95% CI: 1·2 to 7·7). However, the MMR attributable to respiratory causes presented an uptrend of 12·1%/year (95% CI: 8·6 to 15·7) without a join point breaking the trend. One joinpoint was observed for the non-respiratory causes (Figure 2i), with the segment 1990-2009 showing annual growth of 13·2% (95% CI: 10 to 16·5). The AAPC estimated an uptrend of 7·2%/year (95% CI:3·5 to 11·1) for the total period.

### Parallel Interrupted Time Series

**Figure 3:**
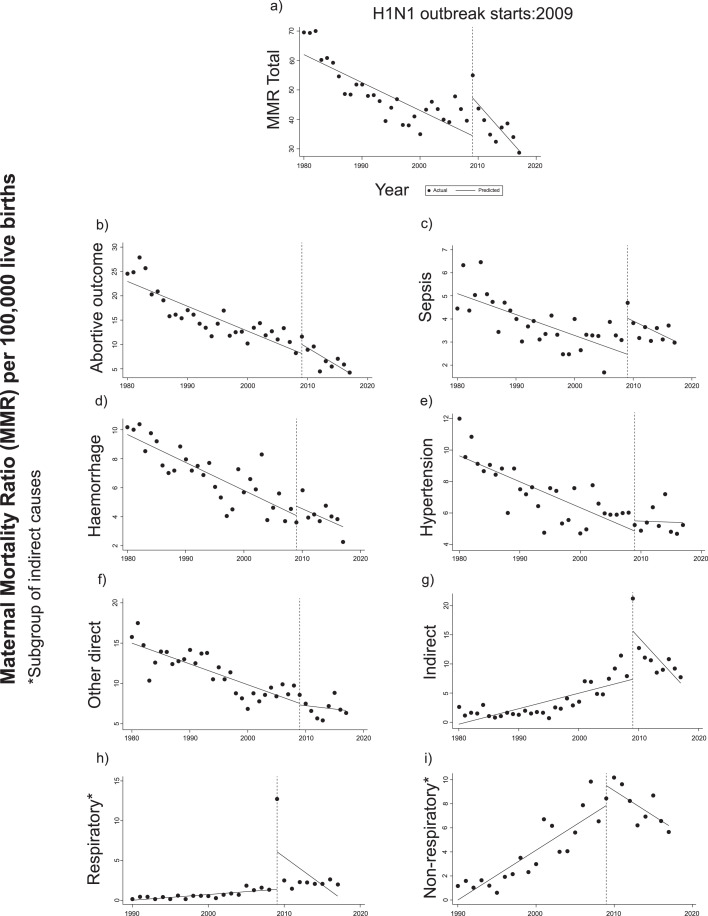
Interrupted time series analyses used to evaluate the effect of the 2009 H1N1 pandemic on the total maternal mortality ratio (a) and maternal mortality ratios due to abortive outcome (b), sepsis (c), haemorrhage (d), hypertension (e), other direct causes (f), total indirect causes (g), respiratory causes (h), and non-respiratory causes (i), using official records from 1980-2017. Vertical lines represent the year for the emergence of the pandemic H1N1 influenza virus. A significant change in level was identified with the pandemic only for a, c, g, and h without evidence of slope change (a, c, h) or reversing in the following years (g), suggesting the outbreak had a transient effect on mortality for these causes.

Parallel ITS analyses of the effect of A[H1N1]pmd09 on the MMR is detailed in Table 2 and plotted in Figure 3. An initial downward trend of 0·94 deaths/100,000 live births per year (95% CI: -1·40 to -0·49) was estimated for the total MMR before the outbreak (Figure 3a). In 2009, a major change in level was identified in the total MMR (12·74; 95% CI: from 4·65 to 20·82/100,000 live births) without evidence of slope change in the following years, suggesting the outbreak had a significant but transient effect on the total MMR.

**Table 2:** Impact of the 2009 H1N1 pandemic on total maternal mortality ratio (MMR) and cause-specific MMR due to abortive outcome, sepsis, haemorrhage, hypertension, other direct causes, and indirect causes, in Argentina, 1980-2017. Interrupted time series analyses.

	**Initial Slope**	**Change in level**	**Slope Change**	**Segment 2009-2017**
	**β_1_ (95% CI**§**); p-value**	**β_2_ (95% CI); p-value**	**β_3_ (95% CI); p-value**	**β_4_ (95% CI); p-value**
**MMR Total**†	-0·94 (-1·40- -0·49); p<0·001	12·74 (4·65-20·82); p=0·003	-1·28 (-2·59-0·32); p=0·056	-2·23 (-3·34- -1·11); p<0·001
**MMR by:**				
**Abortive outcome**	-0·50 (-0·69- -0·31); p<0·001	1·82 (-1·08-4·74); p=0·211	-0·20 (-0·57-0·15); p=0·261	-0·71 (-1·01- -0·41); p<0·001
**Sepsis**	-0·09 (-0·12- -0·05); p<0·001	1·57 (1·02-2·11); p<0·001	-0·03 (-0·14-0·07); p=0·559	-0·12 (-0·21- -0·02); p=0·011
**Haemorrhage**	-0·19 (-0·23- -0·14); p<0·001	0·64 (-0·51-1·80); p=0·264	0·01 (-0·15-0·18); p=0·854	-0·17 (-0·33- -0·01); p=0·032
**Hypertension**	-0·16 (-0·22- -0·09); p<0·001	0·65 (-0·69-2·00); p=0·328	0·14 (0·01-0·28); p=0·036	-0·01 (-0·14- 0·11); p=0·809
**Other direct**	-0·25 (-0·34- -0·17); p<0·001	-0·18 (-1·97-1·60); p=0·834	0·17 (-0·10-0·44); p=0·212	-0·08 (-0·32-0·14); p=0·456
**Indirect**	0·26 (0·13-0·39); p<0·001	8·22 (4·50-11·94); p<0·001	-1·37 (-2·14- -0·61); p=0·001	-1·11 (-1·82- -0·40); p=0·003
**Respiratory**^‡^	0·07 (0·04-0·10); p<0·001	4·73 (0·27-9·18); p=0·038	-0·76 (-1·58-0·05); p=0·066	-0·69 (-1·51-0·11); p=0·090
**Non-respiratory**^‡^	0·41 (0·29-0·53); p<0·001	1·64 (-0·10-3·38); p=0·064	-0·83 (-1·01- -0·64); p<0·001	-0·41 (-0·58- -0·25); p<0·001

† MMR: Maternal Mortality Ratio per 100,000 live births; β1: Pending before the intervention; β2: Level change in period immediately after the start of intervention; β3: Interaction term between the start time of the study and the variable that represents pre and post intervention periods indicating effects over time; β4: Calculated trend of the model by a linear combination of coefficients (β1+ β3)

§ CI: Confidence Interval. ^‡^Subgroup of indirect obstetric causes, period 1990-2017.

The specific MMR attributed to sepsis decreased by 0·09 deaths/100,000 live births for each year before 2009 (95% CI: -0·12 to -0·05). In the year of the outbreak, a change was detected, reaching 1·57 death/100,000 live births (95% CI: 1·02 to 2·11) without evidence of a change in the post-pandemic slope (Figure 3c). In contrast, the MMR attributed to hypertension presented an increasing trend change on the post-pandemic slope, with 0·14 deaths/100,000 live births per year (95% CI: 0·01 to 0·28) without evidence of a level change during the outbreak (Figure 3e).

The MMR attributed to indirect causes before 2009 showed an increasing trend of 0·26 deaths/100,000 live births per year (95% CI: 0·13 to 0·39). A major change in level was observed in 2009, increasing from 0·26 deaths (95% CI: 0·13 to 0·39) to 8·22/100,000 live births (95% CI: 4·50 to 11·94). After 2009, there was a change in the post-pandemic slope, with an estimated annual decrease of 1·37 deaths/100,000 live births (95% CI: -2·14 to -0·61) (Figure 3g).

Finally, the ITS analysis of the MMR specifically attributable to respiratory causes showed an annual increase of 0·07 deaths/100,000 live births in the pre-pandemic period (95% CI: 0·04-0·10) (Figure 3h). A level change in maternal deaths of 4·73/100,000 live births (95% CI: 0·27 to 9·18; p=0.038) was corroborated in 2009, reversing after the outbreak and without evidence of slope change in the following years. The subgroup of MMR attributable to non-respiratory indirect comorbidities did not show a change in level for the A(H1N1)pmd09 outbreak (marginal p-value of 0.064), but the post-pandemic slope reverted to a downward trend (Figure 3i).

## Discussion

In this population-based natural experiment comprising 38-year time-series data from 1980 to 2017, mortality of women during pregnancy, childbirth, and the puerperium, significantly decreased in Argentina (58·6% reduction). Deaths from haemorrhage, sepsis, and abortive outcomes, appear to have contributed the most to this decrease. In contrast, the MMR attributable to indirect causes increased in the same period. Notably, an abrupt interruption in the downward trend in total MMR was corroborated in 2009. Parallel ITS analyses of cause-specific maternal mortality confirmed that A(H1N1)pmd09 had significant short-term effects, transiently increasing maternal deaths from respiratory diseases and sepsis but virtually not impacting the mortality trend from other direct obstetric causes and other indirect non-respiratory comorbidities unrelated to pregnancy.

In Argentina, the relative contribution of different maternal death causes on women reflects a total trend that shows a gradual modification in the epidemiological pattern of mortality known as “obstetric transition”, which is mainly characterised by changes in the predominance of deaths from direct obstetric causes to indirect causes.^16^ In fact, reflecting this epidemiological transition, the MMR attributable to haemorrhage, abortive outcomes, and sepsis exhibited notable reductions throughout the time series analysed, while the MMR attributable to indirect causes tripled over the same timeframe. These findings indicate that the change in context became relevant to the growing number of pregnant women with pre-existing chronic conditions and nutritional diseases,^17^^,^^18^ as well as the decision by women to delay pregnancy.^2^ Additionally, during this obstetric transition, in contrast to the natural events related to pregnancy, components of the institutionalisation of policies and programmes for qualified birth assistance, obstetric emergency care, and healthcare for high-risk pregnancies became more relevant.^2^^,^^16^ According to our study, emerging infectious epidemics appear to have a significant impact on this obstetric transition as well.

The findings in the joinpoint regression indicate that maternal mortality continuously and steadily decreased, with different events implicated at different times. In fact, two joinpoints were noted on the total MMR trend. The first join point, in 1998, reflected the MMR attributable to sepsis. This year coincides with the change in the registry from ICD9 to ICD10, the latter probably showing that the causes of death were underreported in the previous coding system.^12^ The second join point, in 2009 reflected a dual trend change in total MMR and MMR from indirect causes. The 2009 join point is consistent with the ITS results and corroborates the effects of A(H1N1)pmd09 on specific causes of maternal death.

The results of parallel ITS analyses confirmed an acceptable registry sensitivity not only for the total MMR level but also for cause-specific MMR. Complications in the respiratory system and sepsis were associated with changes in mortality/100,000 live births, resulting in a substantial and abrupt increase in total maternal mortality attributable to A(H1N1)pmd09. Most importantly, these findings are in turn relevant for the epidemiological surveillance and comparative analyses of the ongoing SARS-CoV-2 outbreak and any other emerging pandemics in the future. For example, a very recent 10-year time-series study conducted in Brazil (2011 to 2020) reported a significant increase in total maternal mortality with the incidence of the new pandemic SARS-CoV-2 over the last year of data collection^24^. However, this study did not evaluate cause-specific maternal deaths. In our population-based natural experiment with a virtually complete time series of Argentinian vital statistics, we found that the harmful effect of the pandemic H1N1 2009 influenza virus on maternal health, was remarkably restricted to the respiratory system and sepsis. Maternal mortality from non-respiratory comorbidities unrelated to pregnancy along with other multiple direct obstetric causes appeared virtually unaffected by this pandemic virus. Recent epidemiological data from Mexico^25^, suggest that the case would be different for the ongoing SARS-CoV-2 pandemic, with a significant increase in maternal deaths from non-respiratory comorbidities being observed as well. In contrast with the pandemic H1N1 virus, the new pandemic coronavirus might be impacting multiple systems and organs during pregnancy, childbirth, and the puerperium. Further studies using Argentinian cause-specific mortality data before, during, and after the SARS-CoV-2 outbreak may add consistency to these early epidemiological observations.

Systematic reviews^7^, ^8^ and other Latin American reports^11^^,^^12^^,^^19^^,^^20^ confirm an increase in the risk of hospitalization, intensive care unit admission, and death of infected pregnant women during the A(H1N1)pmd09 pandemic compared to the uninfected group. Respiratory diseases play important roles in indirect causes of maternal mortality through complications of pre-existing conditions such as cardiovascular diseases or asthma that could be worsened during serious infection.^21^, ^22^, ^23^ In addition, there may have been delays in early diagnosis because of anatomic and physiological changes during pregnancy that could have masked known symptoms of a respiratory disease.^21^, ^22^ Furthermore, over the course of an emerging pandemic, delays in diagnosis may be accentuated by the interruption of medical services, insufficient availability of laboratory test, and the lack of attendance at health facilities for fear of contagion.^24^, ^25^

Importantly, maternal deaths from sepsis rose sharply during the 2009 H1N1 pandemic. This major regressive effect, albeit apparently transitory, is a red flag considering the great advance in our handling of this ubiquitous threat to maternal health. Recently, similar results with endemic influenza viruses have been reported elsewhere.^26^ During pregnancy, physiological and immunological changes predispose the pregnant woman to systemic infections, which may be aggravated by influenza,^27^^,^^28^ which in turn is more frequent and riskier during pregnancy.^29^ It is important to establish whether the new pandemic coronavirus SARS-CoV-2 may also be increasing maternal mortality from sepsis and whether this complication is prone to endemic variants over time.

The strength of natural experiments from large interrupted time-series is that the effect of a causal factor is captured by controlling for underlaying trends before and after the introduction of an exogenous event. Population-based natural experiments add minimisation of sampling bias under the assumption that the study includes virtually the entire population exposed and non-exposed to risk (e.g., pandemic emerging viruses) and all deaths attributable to exposure over time. Thus, the large numbers accounted by this type of study, allow us to estimate unbiased effect sizes for cause-specific deaths with greater precision.

This natural experiment has some inherent limitations. Because our study is based on audited vital statistics, there is a lag in obtaining final corrected and anonymised data from the official source. Although it was based on complete officially audited vital statistics collected uninterruptedly over 38 years, the potential for underreporting of maternal deaths from national vital statistics is evident, particularly before the ICD-10 was adopted in 1997. Later, an intentional search for maternal deaths among the causes of death was incorporated, improving the completeness of the Argentinian records. In addition, ICD homologation is imperfect, potentially introducing an artefactual change in level with the new coding system. To reduce historical bias, the inclusion of a control trend obtained from a population unexposed to the event of interest is recommended for analysis.^30^ Unfortunately, finding a maternal mortality trend appropriate as a control population (not exposed to A(H1N1)pmd09) was difficult since the virus largely impacted other Latin American countries simultaneously. However, parallel time series of different causes thought to be unaffected by the event of interest over the same exposed population may be utilised as surrogate counterfactual.^13^^,^^30^ For instance, in this study, no evidence of any effect was found in parallel ITS analyses for deaths by non-respiratory indirect causes and abortive outcome (*i.e.* plausible controls for respiratory complications and sepsis, respectively), supporting a cause-effect association.

Finally, in conclusion, major efforts will be needed in early organised healthcare to prevent maternal deaths and monitor the impact of emerging pandemic viruses on women during pregnancy, childbirth, and the puerperium. In the case of Argentina, the mortality record showed an acceptable quality to reflect very specific changes in the MMR caused by an emerging infectious outbreak. In fact, pandemic H1N1 influenza virus contributed to an abrupt and short-term increase in maternal deaths from the respiratory system- and sepsis-related complications, with no evidence of impacting significantly other causes of death. Thus, the Argentinian maternal death record may be useful to evaluate the specific effects of the ongoing SARS-CoV-2 pandemic and any other emerging infectious outbreak in the future.

## Contributors

Project Design: Elard Koch, María Elena Critto, Lenin de Janon, Miguel Bravo, Yordanis Enriquez, and Ruth Weinberg.

Acquisition of data: María Elena Critto, Miguel Bravo, Lenin de Janon, and Ruth Weinberg.

Data analysis and interpretation of data: María Elena Critto, Yordanis Enriquez, and Miguel Bravo.

Manuscript drafting: Elard Koch, María Elena Critto, Yordanis Enriquez, and Miguel Bravo.

Review: Elard Koch, Lenin de Janon, Ruth Weinberg, and Adolfo Etchegaray.

## Declaration of Competing Interest

The authors have no conflict of interest with the results or conclusions of this study.

## Data Availability

The data underlying this article were accessed from DEIS: https://argentina.gob.ar/salud/deis. The derived data generated in this research will be shared at reasonable request to the corresponding author. Additional data are available online as *Supplementary Material*.

## Appendix Supplementary materials

Image, application 1

Image, application 2

Image, application 3

Image, application 4

Image, application 5

Image, application 6

Image, application 7

Image, application 8

## References

[1] Hogan MC, Foreman KJ, Naghavi M (2010). Maternal mortality for 181 countries, 1980–2008: a systematic analysis of progress towards Millennium Development Goal 5. Lancet.

[2] Geller SE, Koch AR, Garland CE, MacDonald EJ, Storey F, Lawton B. (2018). A global view of severe maternal morbidity: moving beyond maternal mortality. Reprod Health.

[3] Murray CJ, Frenk J. (2008). Health metrics and evaluation: strengthening the science. Lancet.

[4] Craig P, Cooper C, Gunnell D (2012). Using natural experiments to evaluate population health interventions: new Medical Research Council guidance. J Epidemiol Community Health.

[5] Somerville LK, Basile K, Dwyer DE, Kok J. (2018). The impact of influenza virus infection in pregnancy. Future Microbiol.

[6] Jain S., Kamimoto L., Bramley A.M. (2009). Hospitalized patients with 2009 H1N1 influenza in the United States, April-June 2009. N Engl J Med.

[7] Mosby LG, Rasmussen SA, Jamieson DJ. (2011). 2009 pandemic influenza A (H1N1) in pregnancy: a systematic review of the literature. Am J Obstet Gynecol.

[8] García-Sancho C, Fernández-Plata R, Martínez-Briseño D (2014). Efecto de la infección por influenza A H1N1 en mujeres embarazadas y en los neonatos en 2009: Revisión de la literatura. Neumol Cir Tórax.

[9] Jamieson DJ, Honein MA, Rasmussen SA (2009). H1N1 2009 influenza virus infection during pregnancy in the USA. Lancet.

[10] ANZIC Influenza Investigators and Australasian Maternity Outcomes Surveillance System (2010). Critical illness due to 2009 A/H1N1 influenza in pregnant and postpartum women: population based cohort study. B*MJ*.

[11] Balanzat AM, Hertlein C, Apezteguia C (2012). An analysis of 332 fatalities infected with pandemic 2009 influenza A (H1N1) in Argentina. PLoS One.

[12] Ministerio de Salud de la Nación Argentina (2021).

[13] Bernal JL, Cummins S, Gasparrini A. (2017). Interrupted time series regression for the evaluation of public health interventions: a tutorial. Int J Epidemiol.

[14] Kim HJ, Fay M, Feuer E, Midthune D. (2001). ‘ Permutation tests for joinpoint regression with applications to cancer rates’. Statistics in Medicine 2000 19:335–351. Stat Med.

[15] Linden A. (2015). Conducting interrupted time-series analysis for single- and multiple-group comparisons. Stata J.

[16] Souza JP, Tunçalp Ö, Vogel JP (2014). Obstetric transition: the pathway towards ending preventable maternal deaths. BJOG.

[17] Kassebaum NJ, Bertozzi-Villa A, Coggeshall MS (2014). Global, regional, and national levels and causes of maternal mortality during 1990-2013: a systematic analysis for the Global Burden of Disease Study 2013. Lancet.

[18] Creanga AA, Berg CJ, Syverson C, Seed K, Bruce FC, Callaghan WM. (2015). Pregnancy-related mortality in the United States, 2006-2010. Obstet Gynecol.

[19] Fajardo-Dolci G, Meljem-Moctezuma J, Vicente-González E (2013). Analysis of maternal deaths in Mexico occurred during 2009. Rev Med Inst Mex Seguro Soc.

[20] Pfitscher LC, Cecatti JG, Haddad SM (2016). The role of infection and sepsis in the Brazilian network for surveillance of severe maternal morbidity. Trop Med Int Health.

[21] Mehta N, Chen K, Hardy E, Powrie R. (2015). Respiratory disease in pregnancy. Best Pract Res Clin Obstet Gynaecol.

[22] Pfitscher LC, Cecatti JG, Pacagnella RC (2016). Severe maternal morbidity due to respiratory disease and impact of 2009 H1N1 influenza A pandemic in Brazil: results from a national multicenter cross-sectional study. BMC Infect Dis.

[23] Brandt JS, Hill J, Reddy A (2021). Epidemiology of coronavirus disease 2019 in pregnancy: risk factors and associations with adverse maternal and neonatal outcomes. Am J Obstet. Gynecol.

[24] de Carvalho RD, Maria da Conceição NC, Teixeira MG (2021). Impact of COVID-19 pandemic on time series of maternal mortality ratio in Bahia, Brazil: analysis of period 2011–2020. BMC Pregnancy and Childbirth.

[25] Mendez-Dominguez N, Santos-Zaldívar K, Gomez-Carro S, Datta-Banik S, Carrillo G. (2021). Maternal mortality during the COVID-19 pandemic in Mexico: a preliminary analysis during the first year. BMC Public Health.

[26] Ali A, Lamont RF. (2019). Recent advances in the diagnosis and management of sepsis in pregnancy. F1000Res.

[27] Bonet M, Souza JP, Abalos E (2018). The global maternal sepsis study and awareness campaign (GLOSS): study protocol. Reprod Health.

[28] Beigi RH. (2017). Emerging infectious diseases in pregnancy. Obstet Gynecol.

[29] Kourtis AP, Read JS, Jamieson DJ. (2014). Pregnancy and infection. N Engl J Med.

[30] Esposti MD, Spreckelsen T, Gasparrini A (2021). Can synthetic controls improve causal inference in interrupted time series evaluations of public health interventions?. Int J Epidemiol.

