# Supplementary material for: Impact of emerging virus pandemics on cause-specific maternal mortality time series: a population-based natural experiment using national vital statistics, Argentina 1980-2017
Source: Lancet Reg Health Am. 2021 Nov 19;6:100116. doi: 10.1016/j.lana.2021.100116 (PMC9904057; doi:10.1016/j.lana.2021.100116)
Supplement: Supplementary file 1 [file mmc1.docx]

**Supplementary Methods**

WHO defines direct obstetric causes of deaths as deaths “resulting from obstetric complications of the pregnant state (pregnancy, labour and the puerperium), and from interventions, omissions, incorrect treatment, or from a chain of events resulting from any of the above” during pregnancy, childbirth or the puerperium (up to 42 days)^1^ and indirect obstetric causes of deaths as deaths “resulting from previous existing disease or disease that developed during pregnancy and not due to direct obstetric causes but were aggravated by the physiologic effects of pregnancy”.^1^

To construct the time series maternal mortality analysis between 1980 and 2017, we considered the cause-specific groups of deaths defined in the ICD-9 as a reference, adapting the WHO subdivision for the causes of maternal deaths in the ICD-10. Thus, the cause-specific maternal mortality groups used in the study period are specified in Supplementary Table S1.

The ICD assigns a category to each mortality cause represented by a three-character numeric or alphanumeric code for systematic registration, analysis, and international comparison. There are two ways to present the codes: the basic list, whose codes include one or more categories to summarize the information in statistical tables, and the expanded or detailed lists, which provide greater disaggregation of the clinical details by category.^2^

For the construction of the cause-specific maternal mortality groups used in this study, four sources were considered (Supplementary Table S2). The 1982 Annual Vital Records Registry reported the first five groups (1st column),^3^ each one according to the ICD-9 basic list. WHO proposed 11 groups in both a basic and detailed list for the ICD-9 and ICD-10 (2nd and 3rd columns, respectively).^4-6^ Moreover, the 1997 Annual Vital Records Registry presented five maternal death groups.^6^

Since the 1980s, maternal deaths in the Annual Vital Records Registry have been available in the form of a basic list, and we considered this information as a basic structure, preserving the categories (or codes) that originally formed the ICD-9 groups. In this way, abortion (ICD-9) or pregnancy terminated in abortion (ICD-10) in this study was called pregnancy with abortive outcome, and the same codes were retained. In the same way, indirect obstetric causes were identified. For cases in which the codes comprising the groups of the Argentina Annual Vital Records Registry and WHO differed, the translations of the translator of the International Classification of Diseases for WHO^7^ were used as a guide, which are presented before each ICD-9 code (the reference code) of four characters and its corresponding ICD-10 code. For example, for the bleeding group, we retained the ICD-9 and ICD-10 categories, adding to the latter five other codes to approximate the equivalence between both versions: code 641 (ICD-9) corresponds to O44.0; O44.1; O45.0; O45.8; O45.9; O46.0; O46.8; O46.9; O67.0; O67.8; and O67.9.

For the hypertension group, ICD-9 code 643 was equivalent to ICD-10 code O21, which was in the group of other direct causes in the 1997 records registry. This change led to its exclusion from the final group and its inclusion in the group of other direct causes. In the study group called sepsis, we retained codes related to sepsis and pregnancy-related infections (codes 670 and 675 in group 394 of the ICD-9) and their equivalents of the ICD-10. Additionally, the resulting codes (O23, O85, O86, and O91) were evaluated using the translations of the ICD-10 to ICD-9 translator to corroborate the categories that maintained the logical and conceptual consistency of this group. The other ICD-9 codes used for direct obstetric causes but not mentioned in previous groups were assigned using the WHO translator’s translations for the group of other direct obstetric causes.

The deaths attributed to indirect obstetric causes were stratified to identify a subgroup of maternal deaths related to the respiratory system that complicate pregnancy, childbirth and the puerperium (ICD-9 codes 648.9 and ICD-10 O99.5). This group was labelled respiratory causes. The other indirect causes (ICD-9 codes 647-648 and ICD-10 O98-O99 codes, excluding 648.9 ICD-9 and O99.5 ICD-10 codes) made up the subgroup of non-respiratory causes. Information on these two categories has been collected since 1990.

**References**

1. World Health Organization. The WHO application of ICD-10 to deaths during pregnancy, childbirth and puerperium: ICD MM. Geneva: WHO, 2012.

2. Sekikawa A, Horiuchi BY, Edmundowicz D et al. A "natural experiment" in cardiovascular epidemiology in the early 21st century. *Heart* 2003;**89**:255–7.

3. Ministerio de Salud de la Nación Argentina (Ministry of Health of the Argentine Nation). Dirección de Estadística e Información en Salud (National Board of Health Statistics). Serie 5. Estadísticas vitales - información básica. Publicación 1982. 2021. <https://www.argentina.gob.ar/salud/deis> (10 may 2021, date last accessed).

4. Ministerio de Salud de la Nación Argentina (Ministry of Health of the Argentine Nation). Dirección de Estadística e Información en Salud (National Board of Health Statistics). Serie 5. Estadísticas vitales - información básica. Publicación 1985. 2021. <https://www.argentina.gob.ar/salud/deis> (10 may 2021, date last accessed).

5. World Health Organization. International Classification of Diseases: ninth revision, basic tabulation list with alphabetical index. Geneva: WHO, 1978.

6. Ministerio de Salud de la Nación Argentina (Ministry of Health of the Argentine Nation). Dirección de Estadística e Información en Salud (National Board of Health Statistics). Serie 5. Estadísticas vitales - información básica. Publicación 1997. 2021. <https://www.argentina.gob.ar/salud/deis> (10 may 2021, date last accessed).

7. World Health Organization, Division of Health Situation and Trend Assessment. International classification of diseases translator: ninth and tenth revisions: user's guide to electronic tables. Geneva: WHO, 1997.
