# Supplementary material for: Impact of emerging virus pandemics on cause-specific maternal mortality time series: a population-based natural experiment using national vital statistics, Argentina 1980-2017
Source: Lancet Reg Health Am. 2021 Nov 19;6:100116. doi: 10.1016/j.lana.2021.100116 (PMC9904057; doi:10.1016/j.lana.2021.100116)
Supplement: Supplementary file 2 [file mmc2.docx]

**Table S1. Homologation** **of International Statistical Classification of Diseases (ICD) Revisions 9 and 10 for cause-specific maternal mortality groups in Argentina, 1980-2017.**

| **Maternal Mortality Groups** | **ICD-9 (1980-1996)** | **ICD-10 (1997-2017)** |
| --- | --- | --- |
|  | **Detailed List** | **Detailed List** |
| Abortive outcome | 630-639 | O00-O07 |
| Sepsis | 670  675 | O23  O85,O86,  O91 |
| Haemorrhage | 640-641, 666 | O20  O44-O46  O67  O72 |
| Hypertension | 642 | O10-O16 |
| Other direct  obstetric causes | 643-646  650-659  660-665  667-669  671-674  676 | O21, O22, O24-O26, O28, O29,  O30-O36, O40-O43, O47, O48,  O60-O66, O68-O71, O73-O75,  O80-O84, O87-O89, O90, O92,  O94, O95,  O96*, O97* |
| Total indirect  obstetric causes | 647, 648 | O98, O99 |
| *Subgroup of indirect causes:* | |  |
|  |  |  |
| Respiratory indirect | 648.9 | O99.5 |
| Non-respiratory indirect | 647, 648+ | O98, O99+ |

*Late maternal deaths were excluded from the maternal mortality ratio calculation.

+Indirect causes of respiratory diseases were excluded from this group.
