# Supplementary material for: Impact of emerging virus pandemics on cause-specific maternal mortality time series: a population-based natural experiment using national vital statistics, Argentina 1980-2017
Source: Lancet Reg Health Am. 2021 Nov 19;6:100116. doi: 10.1016/j.lana.2021.100116 (PMC9904057; doi:10.1016/j.lana.2021.100116)
Supplement: Supplementary file 3 [file mmc3.docx]

**Table S2. Comparison of groups of cause-specific maternal mortality according to different sources**

| **ICD-9**  **DEIS 1982** † | **ICD-9 WHO**  **(1980-1996) §** | | **ICD-10 WHO (1997-2017) §§** | | **ICD-10**  **DEIS 1997** †† | | **Study proposal** | | | |
| --- | --- | --- | --- | --- | --- | --- | --- | --- | --- | --- |
|  |  |  |  |  |  |  | **ICD-9 ICD-10** | | | |
| **Groups (BL)** | **BL** | **DL** | **BL** | **DL** | **Groups** | **DL** | **Groups** | **DL** | **DL** |  |
| Abortion  (380-389) | 380 | 634 | 092 | O00-O07 | Pregnancy with  abortive outcome | O00-O07 | Abortive outcome | 630-639 | O00-O07 |  |
|  | 381 | 635 |  |  |  |  |  |  |  |  |
|  | 382 | 636 |  |  |  |  |  |  |  |  |
|  | 389 | 630-633,  637-639 |  |  |  |  |  |  |  |  |
| Haemorrhage  (390) | 390 | 640-641  666 | 093 | O10-O16,  O20-O26,  O28-O36,  O40-O48,  O60-O75,  O85-O92,  O95 | Antepartum Haemorrhage | O44-O46 | Haemorrhage | 640-641  666 | O20  O44-O46  O67  O72 |  |
|  |  |  |  |  | Postpartum Haemorrhage | O72 |  |  |  |  |
| Toxaemia  (391) | 391 | 642, 643 |  |  | Hypertensive disorders | O10-O16 | Hypertension | 642 | O10-O16 |  |
| Other direct obstetric causes  (392-399) | 393 | 660 |  |  |  |  |  |  |  |  |
|  | 394 | 670-676 |  |  | Sepsis and other complications of the puerperium | O85-O92 | Sepsis | 670  675 | O23  O85, O86,  O91 |  |
|  | 399 | 644-646,  651-659,  661-665,  667-669 | 094 | O96,O97 | Other direct causes | O20-O43,  O47,O48,  O60-O71,  O73-O75,  O95 | Other direct obstetric causes | 643-646  650-659  660-665  667-669  671-674  676 | O21, O22, O24-O26, O28, O29, O30-O36, O40-O43,O47, O48, O60-O66, O68-O71,  O73-O75, O80-O84,  O87-O89, O90, O92, O94,O95, O96*,O97* |  |
| Indirect  obstetric causes (409) | 409 | 647, 648 | 095 | O98,O99 | Indirect  obstetric causes | O98, O99 | Total Indirect obstetric causes | 647, 648 | O98, O99 |  |
|  | 419 | 650 |  |  |  |  | Respiratory indirect causes | 648.9 | O99.5 |  |
|  |  |  |  |  |  |  | Non-  respiratory | 647, 648+ | O98, O99+ |  |

DEIS: In English, National Board of Health Statistics; BL: Basic List; DL: Detailed List; †Maternal deaths groups from the Argentine official records,1982; **§**World Health Organization (WHO), Correspondence Code List between the basic list for tabulation and the detailed list of three-digit categories of the International Classification of Diseases 9 Revision (ICD -9), 1975; **§§**Correspondence Code List between the basic list for tabulation and the detailed list of three-digit categories of the ICD 10 Revision (ICD -10); ††Maternal deaths groups from the Argentine official records, 1997. +Indirect causes of respiratory diseases were excluded. *Late maternal deaths were excluded from the MMR calculation.
