# Supplementary material for: Impact of emerging virus pandemics on cause-specific maternal mortality time series: a population-based natural experiment using national vital statistics, Argentina 1980-2017
Source: Lancet Reg Health Am. 2021 Nov 19;6:100116. doi: 10.1016/j.lana.2021.100116 (PMC9904057; doi:10.1016/j.lana.2021.100116)
Supplement: Supplementary file 4 [file mmc4.docx]

**Table S3. Percentage changes in the total maternal mortality ratio (MMR) and cause-specific MMRs by group, Argentina 1980-2017**

|  |  |  | **Maternal Mortality Ratio (MMR) from specific casues** | | | | | | | | |
| --- | --- | --- | --- | --- | --- | --- | --- | --- | --- | --- | --- |
| **Year** | **MMR total** | **Direct** | Direct | | | | | **Indirect** | Indirect | | |
|  |  |  | abortive outcome | sepsis | haemorrhage | hypertension | other direct |  | respiratory | non-respiratory | |
| 1980 | 69·5 | 67·0 | 24·5 | 4·4 | 10·2 | 12·0 | 15·8 | 2·6 | ·· | ·· | |
| 1990 |  |  |  |  |  |  |  |  | 0·1 | 1·2 | |
| 2017 | 28·8 | 21·1 | 4·3 | 3·0 | 2·3 | 5·3 | 6·4 | 7·7 | 2·0 | 5·7 | |
| Change (*) | -40·7 | -45·8 | -20·3 | -1.5 | -7·9 | -6·8 | -9·4 | 5·1 | 1·8 | 4·5 | |
| Change (%) | -58·6 | -68·4 | -82·6 | -32·9 | -77·7 | -56·4 | -59·5 | 197 | 1248·4 | 381·6 | |
| *maternal mortality ratio per 100,000 live births. | | | | | | | | | | |  |
