# Supplementary material for: Impact of emerging virus pandemics on cause-specific maternal mortality time series: a population-based natural experiment using national vital statistics, Argentina 1980-2017
Source: Lancet Reg Health Am. 2021 Nov 19;6:100116. doi: 10.1016/j.lana.2021.100116 (PMC9904057; doi:10.1016/j.lana.2021.100116)
Supplement: Supplementary file 5 [file mmc5.docx]

**
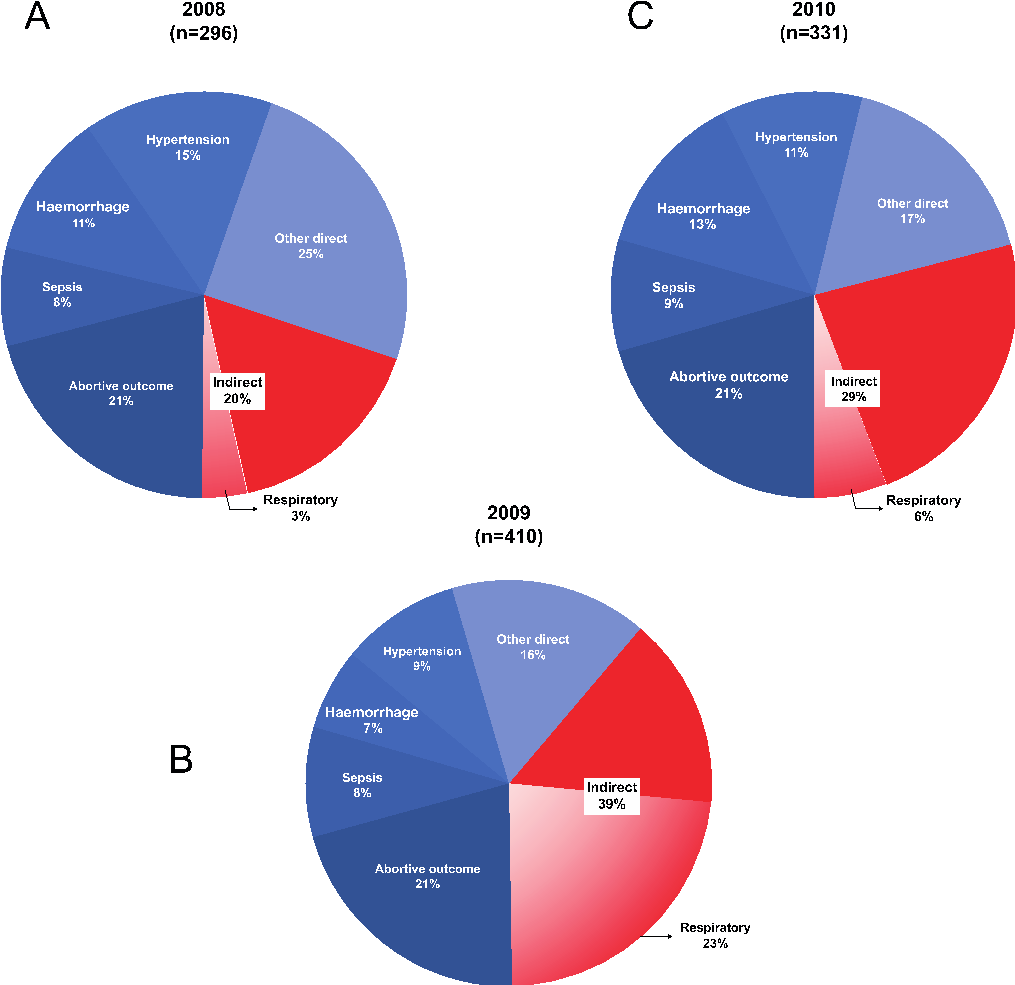
**

**Figure S1. Relative importance of cause-specific maternal mortality groups in Argentina in 2009 and in the years before and after the pandemic**

Cause-specific maternal mortality groups corresponding to pregnancy and abortive outcome, sepsis, haemorrhage, hypertension, other direct obstetric causes, indirect obstetric and indirect causes attributable to respiratory diseases. (see Supplementary Table S1 for the ICD codes used)
