# Supplementary material for: Impact of emerging virus pandemics on cause-specific maternal mortality time series: a population-based natural experiment using national vital statistics, Argentina 1980-2017
Source: Lancet Reg Health Am. 2021 Nov 19;6:100116. doi: 10.1016/j.lana.2021.100116 (PMC9904057; doi:10.1016/j.lana.2021.100116)
Supplement: Supplementary file 6 [file mmc6.docx]

**Caption for supplementary material**

Captions (suggested)

- Supplementary Methods
- Table S1. Homologation for cause-specific maternal mortality groups in Argentina according to the International Statistical Classification of Diseases (ICD) Revisions 9 and 10.
- Table S2. Construction of cause-specific groups for maternal mortality according to different sources.
- Table S3. Percentage changes in the total maternal mortality ratio (MMR) and cause-specific MMRs, Argentina 1980-2017
- Figure S1. Relative importance (% from total maternal deaths) of cause-specific maternal mortality groups in Argentina in 2009 and in the years before and after the pandemic.
- Spanish abstract
- Portuguese abstract

Submitted supplementary files

- TLRHAMERICAS-D-21-00171 – Supplementary methods
- TLRHAMERICAS-D-21-00171 – Supplementary Table S1
- TLRHAMERICAS-D-21-00171 – Supplementary Table S2
- TLRHAMERICAS-D-21-00171 – Supplementary Table S3
- TLRHAMERICAS-D-21-00171 – Supplementary Figure S1
- TLRHAMERICAS-D-21-00171 – Spanish abstract
- TLRHAMERICAS-D-21-00171 – Portuguese abstract
