# Supplementary material for: Impact of emerging virus pandemics on cause-specific maternal mortality time series: a population-based natural experiment using national vital statistics, Argentina 1980-2017
Source: Lancet Reg Health Am. 2021 Nov 19;6:100116. doi: 10.1016/j.lana.2021.100116 (PMC9904057; doi:10.1016/j.lana.2021.100116)
Supplement: Supplementary file 7 [file mmc7.docx]

**Resumo**

**Antecedentes:** Os vírus pandêmicos emergentes podem ter vários efeitos nocivos sobre a saúde materna. Este estudo examina os efeitos do vírus da gripe influenza pandêmica em séries temporais de mortalidade materna por causas específicas, usando estatísticas vitais argentinas.

**Métodos:** Conduzimos uma pesquisa natural de base populacional usando registros vitais nacionais de mortes maternas entre 1980 e 2017. Modelos de regressão de pontos de junção foram usados para modelar séries temporais da razão de mortalidade materna (RMM). A capacidade do registro para detectar os efeitos do vírus da gripe pandêmica H1N1 2009 na RMM de causa específica foi analisada usando um painel de séries temporais interrompidas (STI).

**Resultados:** Durante este estudo de 38 anos, a RMM diminuiu 58·6% (69·5 a 28·8 mortes / 100.000 nascidos vivos), passando de causas obstétricas diretas (67·0 a 21·1 / 100.000 nascidos vivos; 68·4% de redução) para causas indiretas (2·6 a 7·7 / 100.000 nascidos vivos; 196·2% de incremento). A análise de regressão mostrou uma redução média de

-2·2% /ano (IC 95%: -2·9 a -1·4) com 2 pontos de inflexão na tendência total (1998 e 2009). As análises de STI revelaram que o vírus A H1N1 pandêmico teve um efeito crescente na mortalidade por complicações do sistema respiratório e relacionadas à sepse (mudança de nível 4·7 e 1·6 / 100.000 nascidos vivos, respectivamente), revertendo após o surto. Nenhum efeito foi encontrado na RMM devido a distúrbios hipertensivos, hemorragia, aborto, outras causas obstétricas diretas e comorbidades indiretas não respiratórias.

**Interpretação:** O registro de óbitos maternos da Argentina parece eficaz para a detecção de diferentes efeitos devido a epidemias infecciosas emergentes sobre a saúde materna. Em um experimento natural de base populacional, o vírus pandêmico A H1N1 afetou a mortalidade materna quase exclusivamente por complicações relacionadas ao sistema respiratório e a sepse.

**Financiamento:** Apoiado pela FISAR www.fisarchile.org
