# Supplementary material for: Impact of emerging virus pandemics on cause-specific maternal mortality time series: a population-based natural experiment using national vital statistics, Argentina 1980-2017
Source: Lancet Reg Health Am. 2021 Nov 19;6:100116. doi: 10.1016/j.lana.2021.100116 (PMC9904057; doi:10.1016/j.lana.2021.100116)
Supplement: Supplementary file 8 [file mmc8.docx]

**Resumen

Antecedentes:** Los virus pandémicos emergentes pueden tener múltiples efectos nocivos sobre la salud materna. Este estudio examina los efectos de un virus de influenza pandémica mediante series de tiempo ininterrumpidas de mortalidad materna por causas específicas, utilizando estadísticas vitales argentinas.

**Métodos:** Realizamos un experimento natural con base poblacional a partir de registros vitales nacionales de muertes maternas entre 1980 y 2017. Se utilizaron modelos de regresión con punto de unión para modelar series de tiempo de la razón de mortalidad materna (RMM). La sensibilidad del registro para detectar los efectos del virus de la influenza pandémica A H1N1 2009 sobre las causas específicas de la RMM se analizó mediante un panel de series de tiempo interrumpidas (STI).

**Hallazgos:** Durante este estudio de 38 años, la RMM disminuyó en un 58·6% (69·5 a 28·8 muertes/100.000 nacidos vivos), pasando de causas obstétricas directas (67·0 a 21·1/100.000 nacidos vivos; 68·4% de disminución) a causas indirectas (2·6 a 7·7/100.000 nacidos vivos; 196·2% de aumento). El análisis de regresión mostró una reducción promedio de -2·2%/año (IC 95%: -2·9 a -1·4) con 2 puntos de inflexión en la tendencia total (1998 y 2009). Los análisis de STI revelaron que el virus pandémico A H1N1 tuvo un efecto creciente sobre la mortalidad por complicaciones del sistema respiratorio y relacionadas con la sepsis (cambio de nivel 4·7 y 1·6/100.000 nacidos vivos, respectivamente), revirtiéndose después del brote. No se encontró ningún efecto sobre la RMM por trastornos hipertensivos, hemorragia, desenlace abortivo, otras causas obstétricas directas y comorbilidades indirectas no respiratorias.

**Interpretación:** El registro de defunciones maternas de Argentina parece sensible para detectar diferentes efectos de las epidemias infecciosas emergentes sobre la salud materna. En el experimento natural poblacional, el virus pandémico A H1N1 afectó la mortalidad materna casi exclusivamente por complicaciones relacionadas con el sistema respiratorio y la sepsis.

**Financiamiento:** apoyado por FISAR www.fisarchile.org
